# Silencing *GmBIR1* in Soybean Results in Activated Defense Responses

**DOI:** 10.3390/ijms23137450

**Published:** 2022-07-05

**Authors:** Dan-Dan Liu, Hu-Jiao Lan, Hashimi Said Masoud, Mei-Yan Ye, Xian-Yong Dai, Chen-Li Zhong, Sheng-Nan Tian, Jian-Zhong Liu

**Affiliations:** 1 Institute of Plant Genetics and Developmental Biology, College of Chemistry and Life Sciences, Zhejiang Normal University, Jinhua 321004, China; dandanliuxy@163.com (D.-D.L.); 18659351751@163.com (H.-J.L.); s.masoud.hashimi@gmail.com (H.S.M.); a19557861320@163.com (M.-Y.Y.); q1178261391@163.com (X.-Y.D.); 18170141671@163.com (C.-L.Z.); xiaotian12260606@163.com (S.-N.T.); 2 Zhejiang Provincial Key Laboratory of Biotechnology on Specialty Economic Plants, Zhejiang Normal University, Jinhua 321004, China

**Keywords:** *Glycine max*, virus-induced gene silencing (VIGS), receptor-like kinases, immune responses, BIR1, salicylic acid

## Abstract

Receptor-like kinases (RLKs) are a large group of pattern recognition receptors (PRRs) and play a critical role in recognizing pathogens, transducing defense signals, and mediating the activation of immune defense responses. Although extensively studied in the model plant Arabidopsis, studies of RLKs in crops, including soybean, are limited. When a *BAK1-interacting receptor-like kinase* (*BIR1*) homolog (referred to as *GmBIR1* hereafter) was silenced by the BPMV (*Bean pod mottle virus*)-induced gene silencing (BPMV-VIGS), it resulted in phenotypes that were reminiscent of constitutively activated defense responses, including a significantly stunted stature with observable cell death on the leaves of the silenced plants. In addition, both SA and H_2_O_2_ were over-accumulated in the leaves of the *GmBIR1*-silenced plants. Consistent with this autoimmune phenotype, *GmBIR1*-silenced plants exhibited significantly enhanced resistance to both *Pseudomonas syringae pv. glycinea* (*Psg*) and *Soybean mosaic virus* (SMV), two different types of pathogens, compared to the vector control plants. Together, our results indicated that *Gm*BIR1 is a negative regulator of immunity in soybean and the function of BIR1 homologs is conserved in different plant species.

## 1. Introduction

Pathogen-associated molecular patterns (PAMPs) are conserved microbial components that can be perceived by plasma membrane-localized pattern recognition receptors (PRRs) and trigger immunity [1]. The *PRRs* constitute the largest gene family (>600 genes) in Arabidopsis [2]. The PRRs include two types of protein families: the receptor-like kinases (RLKs) and receptor-like proteins (RLPs). While a typical RLK contains an extracellular leucine-rich repeat (LRR) domain, a transmembrane domain (TM), and a cytoplasmic kinase domain, an RLP contains only LRR and TM domains but lacks a cytoplasmic kinase domain. The LRR domain is responsible for perception of PAMPs or ligands, and the cytoplasmic kinase domain is involved in intracellular signal transduction [3]. The lack of a kinase domain in a RLP suggests that an RLP needs to pair with RLKs or another cytoplasmic kinase to transduce signals [3].

Some RLKs have been reported to sense PAMPs either from bacteria or fungi and transduce defense signals [4]. The best studied examples are FLAGELLIN SENSING2 (FLS2) and EF-TU RECEPTOR (EFR). FLS2 recognizes the 22 amino acid peptide flg22 from bacterial flagellin, while EFR recognizes an 18 amino acid peptide, elf18, derived from bacterial translation elongation factor EF-TU [5,6]. BAK1 was originally identified as a BRI1 interacting protein, and it forms a protein complex with BRI1 during perception of Brassinosteroids (BRs) [7,8]. It is now clear that BAK1 serves as a common co-receptor for different RLKs or even RLPs that sense ligands ranging from BRs to PAMPs. Upon ligand or PAMP recognition, BAK1 is recruited to form a complex with RLKs such as FLS2, EFR and CERK1 [9,10,11]. The ligand-induced RLKs–BAK1 complex formation initiates dynamic trans-phosphorylation between the receptor, co-receptor, and receptor-like cytoplasmic kinases (RLCKs) such as BOTRYTIS-INDUCED KINASE1 (BIK1) [12,13], thus leading to the activation of downstream signaling.

Using isotope-coded affinity tag reagents and mass spectrometry, Gao et al. [14] identified BAK1 as BIR1-interacting protein on the plasma membrane (PM), which was subsequently confirmed by BiFC and Co-IP assays [14]. BIR1 belongs to the LRRX group of RLKs, with 620 amino acids and 5 LRRs. Compared to RLKs such as FLS2 or BAK1, BIR1 is a smaller protein with a significantly reduced number of LRR repeats [14]. Loss function of *BIR1* in Arabidopsis leads to extensive cell death, induction of *PR* genes, enhanced accumulation of SA and H_2_O_2_ and impairment in the activation of MPK4 by flagellin [14]. Genetic studies show that the *bir1* mutant phenotype is dependent on BAK1, EDS1, and PAD4 and loss-of-function mutations in these genes can partially suppress the autoimmune phenotype of *bir1* mutant, indicating that over-accumulation of SA is partially responsible for the autoimmune phenotype [14,15]. The kinase activity of BAK1 is required for its function in activation of defense responses in *bir1-1*.

SOBIR1 (suppressor of BIR1) was identified as a suppressor of *bir1-1* autoimmune phenotypes in a suppressor screening. Loss of function in SOBIR1 strongly suppresses cell death in *bir1-1*. Combining the *sobir1* and *pad4-1* mutations leads to suppression of all mutant phenotypes of *bir1-1*, suggesting that SOBIR1 functions in parallel with PAD4. SOBIR1 encodes an RLK with four extracellular LRRs and a cytoplasmic kinase domain [14]. BAK1 interacts with SOBIR1 only when BIR1 is absent [15,16,17]. In the absence of BIR1, inhibition of BAK1 by BIR1 is released and BAK1 forms an active receptor complex with SOBIR1, leading to cell death and defense responses [15]. In addition, BAK1 associates with the BIR1 and the copain-like proteins BONZAI1 (BON1)–BON3, which are required to suppress autoimmunity [18].

BIR2 is an inactive pseudokinase and a substrate of BAK1. It forms a constitutive complex with BAK1 to prevent it from interacting with FLS2. The kinase activity of BAK1 is required for BIR2-BAK1 interaction [19]. BIR2 Is released from BAK1 after PAMP treatment and BIR2 controls BAK1-FLS2 complex formation in a ligand-dependent manner [19]. *bir2* mutant plants exhibit enhanced pathogen-associated molecular pattern (PAMP) responses but do not have the dwarf morphology like *bir1-1*. Unlike BIR2, BIR1 is an active kinase, and kinase activity is at least partially required for its function. flg22 treatment leads to dissociation of BIR2 from BAK1 and over-expression of BIR2 attenuates PAMP-triggered responses [19].

Although extensively studied in the model plant Arabidopsis, the investigations of RLKs in crop plants are limited. Using the BPMV-VIGS system, we have recently identified multiple positive and negative regulators of defense responses in soybean [20,21,22,23,24,25]. Here, using the same strategy, we identified *Gm*BIR1 as a negative regulator of defense responses in soybean. Silencing *GmBIR1* led to spontaneous cell death and constitutively activated defense responses. These autoimmune responses are highly correlated with the over-accumulation of both SA and H_2_O_2_. Consistent with the autoimmune phenotypes, the *GmBIR1*-silenced plants displayed a significantly increased resistance to both *Soybean mosaic virus* (SMV) and *Pseudomonas syringae pv. glycinea* (*Psg*). Taken together, our results indicate that *Gm*BIR1 plays a negative regulatory role in soybean immunity, and the function of BIR1 homologs is conserved across plant species. 

## 2. Results

### 2.1. Silencing Glyma.18g246400 Results in Stunted Stature and Spontaneous Cell Death in Soybean

**Figure 1:**
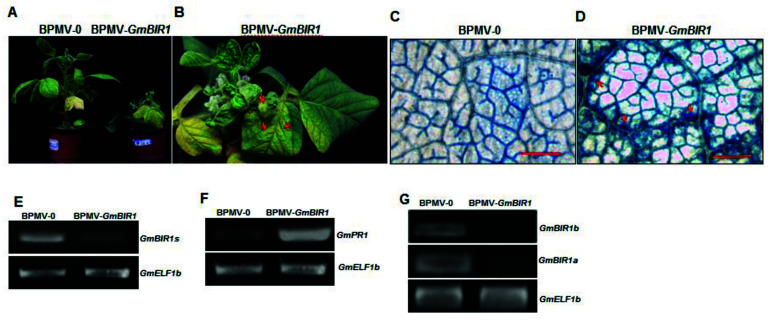
Silencing *GmBIR1* causes both local and systemic hypersensitive response (HR) cell death in soybean. (**A**) The phenotypes of vector control (BPMV-0) and *GmBIR1*-silenced (BPMV-*GmBIR1*) plants at 15 days post infection (dpi); (**B**) Massive cell death was observed on the systemic leaves of *GmBIR1*-silenced plants at 25 dpi. Red arrows point to the dead leaves; (**C**,**D**) Trypan Blue-stained systemic leaves from vector control (**C**) and *GmBIR1*-silenced plants (**D**). Red arrows point to the dead regions. Bar = 500 μm; (**E**) RT-PCR result indicates that the *GmBIR1* transcript level was reduced in *GmBIR1*-silenced plants relative to the vector control plants; (**F**) RT-PCR result indicates that the *GmPR1* transcript level was increased in *GmBIR1*-silenced plants relative to the vector control plants; (**G**) RT-PCR result indicates that the *GmBIR1a and GmBIR1b* transcript level were both reduced in *GmBIR1*-silenced plants relative to the vector control plants. Each experiment was repeated at least twice. Each experiment consisted of 3 replicates and at least 3 plants were used in each replicate.

Through BPMV-VIGS silencing approach, we successfully identified both positive and negative regulators of defense responses in soybean [20,21,22,23,24,25]. Using the same strategy, we found that when *Glyma*.18g246400 was silenced, a significantly stunted stature (Figure 1A) and spontaneous cell death on the systemic leaves were observed for the silenced plants (Figure 1B). The cell death was further confirmed by Tryplan blue staining, which specifically stains the dead cells. The massive blue patches were observed along the lager veins on the systemic leaves of the silenced plants but not on that of vector control plants (Compare Figure 1C,D). RT-PCR analysis confirmed that the transcript level of *Glyma*.18g246400 was significantly reduced in the silenced plants (Figure 1E). Stunted stature and spontaneous cell death are the signature characteristics of the constitutively activated defense responses. Consistent with this, a significantly induced expression of the *PR1* gene was observed in the silenced plants (Figure 1F). These results indicated that *Glyma*.18g246400 encodes a negative regulator of cell death and defense responses. Blast search with the full-length cDNA of *Glyma*.18g246400 revealed that there is a duplicated gene in the soybean genome, *Glyma*.09g246600, which shares 95% identity with *Glyma*.18g246400. It has been reported that VIGS can simultaneously silence genes with a homology greater than 85% at the nucleotide level [26,27]. We confirmed that the silenced phenotype observed for the *Glyma*.18g246400-silenced plants was actually a result of co-silencing both *Glyma*.18g246400 and *Glyma*.09g246600 (Figure 1G). Both *Glyma*.18g246400 and *Glyma*.09g246600 share the highest homology with Arabidopsis *BIR1* (65%), we thus referred to these two genes as *GmBIR1a* and *GmBIR1b*, respectively. Because the *bir1* mutant in Arabidopsis displays a constitutive activated defense phenotype (Gao et al., 2009), our results indicate that the function of BIR1 homologs is conserved in different plants species.

### 2.2. Both SA and H_2_O_2_ Are Over-Accumulated in the GmBIR1-Silenced Plants

**Figure 2:**
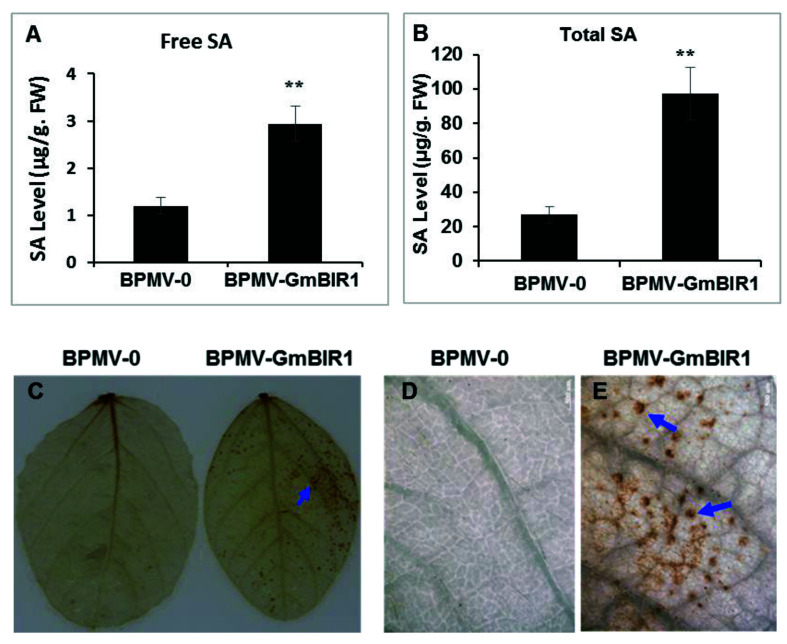
Elevated accumulation of SA and H_2_O_2_ in *GmBIR1*-silenced plants. (**A**,**B**) Free SA (**A**) and Total SA (**B**) levels were quantified in *GmBIR1*-silenced and BPMV-0 empty vector control plants at 20 days post BPMV inoculation. Error bars represent SD for three independent samples. Asterisks indicate significant differences from the control (**, *p* < 0.01, Student’s *t* test). FW, fresh weight. (**C**) Presence of H_2_O_2_ in soybean leaves visualized by staining with DAB. (**D**,**E**) The images taken under the stereomicroscope from part of the regions shown in C, Bar = 500 μm. Oxidized DAB formed a reddish-brown deposit. (Examples of these deposits are indicated by the blue arrows).

SA is a key hormone that positively regulates defense responses against biotrophic pathogens and is over-accumulated in the plants with activated defense responses [20,28]. To examine whether SA over-accumulation occurred in *GmBIR1*-silenced plants, both free SA and bound SA were quantified, and the levels of free SA and bound SA were 2.46- and 4.1-fold higher in the *GmBIR1*-silenced plants than in BPMV-0 control plants, respectively (Figure 2A,B). The over-accumulated level of SA suggests that SA is involved in the constitutively activated defense responses observed in *GmBIR1*-silenced plants.

H_2_O_2_ is a potent trigger of cell death [29,30] and H_2_O_2_ is over-accumulated in the plants with activated defense responses [20,23]. To examine if H_2_O_2_ is over-accumulated in the *GmBIR1*-silenced plants, the leaves collected from both vector control plants and the *GmBIR1*-silenced plants were stained with 3,3-diaminobenzidine (DAB) [31,32]. More intense brown spots were observed on the leaves of the *GmBIR1*-silenced plants compared to the BPMV-0 control plants (Figure 2C), indicating that the cell death observed in the *GmBIR1*-silenced plants was correlated with increased level of H_2_O_2_ accumulation.

### 2.3. Silencing GmBIR1 Enhances Resistance against SMV and Pseudomonas syringae pv. glycinea (Psg), R4 Strain

**Figure 3:**
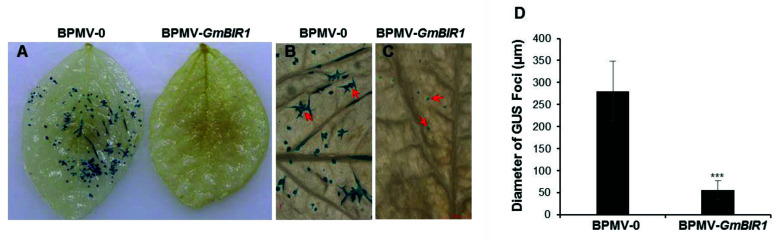
Silencing *GmBIR1* enhances the resistance of soybean plants to SMV. At 18 dpi with BPMV-0 or BPMV-*GmBIR1*, SMV-N-GUS was biolistically delivered onto the detached leaves of silenced and non-silenced plants. At 5 dpi with SMV-N-GUS, the replication and movement of SMV-N-GUS in the biolistically inoculated leaves was detected by GUS staining. The GUS foci were counted and measured. (**A**) The distribution of SMV-N-GUS foci on the leaves of BPMV-0 and BPMV-*GmBIR1* plants. (**B**,**C**) Infection foci of SMV-N-GUS on the leaves of BPMV-0 and BPMV-*GmBIR1* plants. Bar = 2000 μm. (**D**) Comparison of the diameters of SMV-N-GUS foci on the leaves of BPMV-0 and BPMV-*GmBIR1* plants. Error bars represent SD of the diameters of at least 30 GUS foci measured on each of three independent leaves (at least 90 foci). Asterisks indicate a significant difference from the control (***, *p* < 0.001, Student’s *t* test). This experiment was repeated 3 times with similar results.

Activated defense responses are usually correlated with enhanced disease resistance [20,21,22,23,24,25]. To examine whether disease resistance is enhanced in *GmBIR1*-silenced plants, SMV strain N tagged with the GUS marker protein (SMV-N-GUS) [33] was inoculated via biolistic bombardment onto three individual leaves collected from both the vector control plants and the *GmBIR1*-silenced plants. Both the size of the GUS foci and the intensity of GUS staining reflect viral infectivity. At 5 days post inoculation (dpi), the SMV-N-GUS infection was visualized by GUS staining. The GUS infection foci were easily seen on the vector control leaves (Figure 3A, left panels; Figure 3B). However, the GUS spots on the *GmBIR1*-silenced plants were much smaller and only visible under a dissecting microscopy (Figure 3C). The average diameter of the GUS foci formed on the vector control leaves was four-fold of those formed on the *GmBIR1*-silenced leaves (Figure 3D). This result indicates that silencing *GmBIR1* leads to increased resistance against SMV.

**Figure 4:**
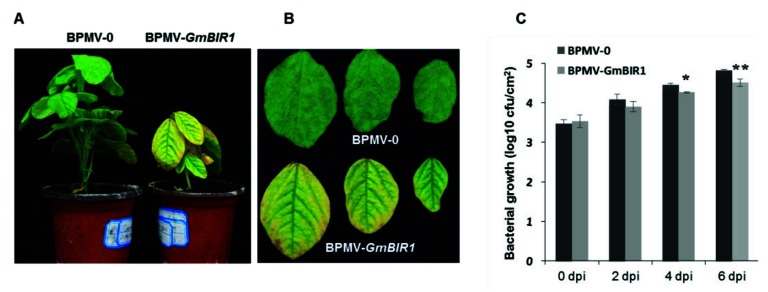
Silencing *GmBIR1* enhanced the resistance of soybean plants to *Pseudomonas syringae pv. glycinea* (*Psg*) R4. (**A**) Comparison of the vector control plants and the *GmBIR1*-silenced plants 4 days after spraying *Psg* suspension; (**B**) Comparison of the leaves from the vector control plants and the *GmBIR1*-silenced plants 4 days after spraying *Psg*; (**C**) Comparison of the colony forming unit (cfu)between the vector control plants and the *GmBIR1*-silenced plants after different days of *Psg* infection. * and ** represent *p* < 0.05 and 0.01, respectively (Student’s *t*-test). This experiment was repeated 2 times with similar results.

We reported that silencing *GmFLS2* compromises soybean resistance against *Pseudomonas syringae pv. glycinea* (*Psg*) R4 [24]. To test the effect of *GmBIR1* silencing on *Psg* infection, we inoculated the BPMV-0 control plants and the *GmBIR1*-silenced plants with *Psg*. At 4- and 6-dpi, the colony forming unit of *Psg* on the vector control leaves was significantly higher than on that of the *GmBIR1*-silenced leaves (Figure 4). Together, these results clearly show that silencing *GmBIR1* enhances resistance of soybean plants against two totally different types of pathogens, SMV and *Psg*.

### 2.4. The Activated Defense Responses in GmBIR1-Silenced Plants Are Independent of GmMPK3/6 Activation

Activation of MAPK signaling pathway is one of the signature hallmarks of activated defense responses [34]. To test whether the activated MAPK signaling pathway contributes to the activated defense responses and enhanced disease resistance observed in *GmBIR1*-silenced plants (Figure 1, Figure 2, Figure 3 and Figure 4), the activation of *Gm*MPK3 and *Gm*MPK6 in response to flg22 was examined both in vector control plants and *GmBIR1*-silenced plants using the Phospho-p44/42 MAP Erk1/2 antibody that recognizes phosphorylation of Arabidopsis and soybean MPK3/4/6 at the sites corresponding to threonine (T) 202/tyrosine (Y) 204 of human (*Homo sapiens*) p44 MAPK [23,24,35]. Contrary to our expectation, activation of both *Gm*MPK3 and *Gm*MPK6 by flg22 was significantly reduced in *GmBIR1*-silenced plants compared to vector control plants (Figure 4), indicating that the activated defense responses observed in the *GmBIR1*-silenced plants are independent of *Gm*MPK3/6 activation.

### 2.5. GmBIR1 Is Localized at the Plasma Membrane (PM) and in the Nucleus

**Figure 5:**
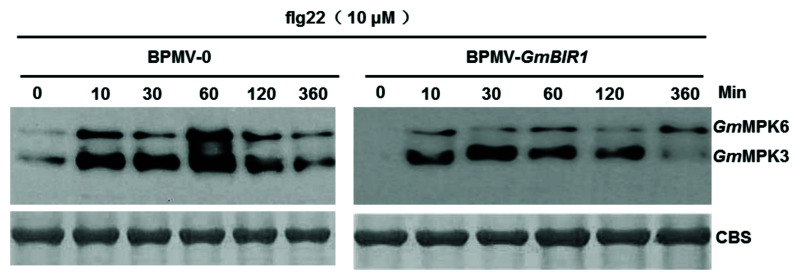
Silencing *GmBIR1* reduces the activation of *Gm*MPK3/*Gm*MPK6 in response to flg22 treatment. Leaf discs from indicated soybean plants were incubated on moist filter paper for 24 h to allow recovery from wounding before being treated with 10 μM flg22 or diluted DMSO. The kinase activities were detected by Western blotting using Phosph-p44/p42 MAP Erk1/2 antibody. Coomassie Blue–stained gel (CBS) used as loading controls. The experiment was repeated 3 times with similar results.

**Figure 6:**
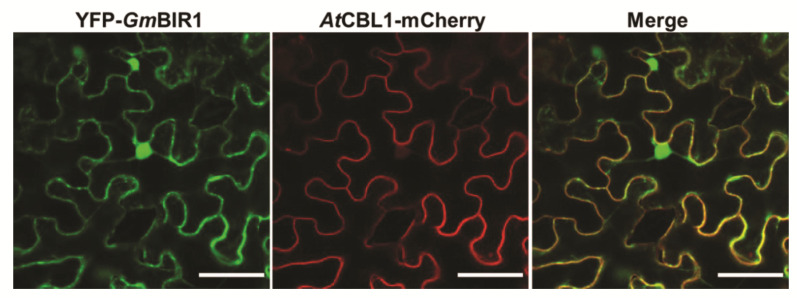
The subcelluar localization of GFP-*Gm*BIR1. The GFP-*Gm*BIR1 localizes both at plasma membrane (PM) and in the nucleus. The PM-localized protein *At*CBL1-mCherry was used as a control. Bar = 50 μm. This experiment was repeated 3 times with similar results.

RLKs normally function at the PM. To investigate the subcellular location of *Gm*BIR1, the full-length *GmBIR1a* was fused to the C-terminus of GFP and transiently co-expressed in *N. benthamiana* leaves with a PM marker, *At*CBL1-mCherry, via agroinfiltration. As shown in Figure 5 and Figure 6, GFP-*Gm*BIR1 was not only co-localized with *At*CBL1-mCherry but also presented in the nucleus, indicating that *Gm*BIR1 has dual subcellular localizations at the PM and in the nucleus.

## 3. Discussion

### 3.1. The Function of BIR1 Homologs Is Conserved from Different Plant Species

It has been reported that the extensive cell death, induction of *PR* genes, enhanced accumulation of SA and H_2_O_2_, impaired activation of MPK4, and enhanced resistance to different pathogens were observed for Arabidopsis *bir1-1* mutant [14,15,36,37,38]. Similarly, spontaneous cell death, constitutively activated autoimmune responses (including induced *PR* gene expression), accumulated levels of both SA and H_2_O_2,_ and enhanced disease resistance to both viral and bacterial pathogens were also observed for the *GmBIR1*-silenced soybean plants (Figure 1, Figure 2 and Figure 3). The phenotypic reminiscence between *bir1-1* mutant and the *GmBIR1*-silenced plants strongly indicate that the functions of BIR1 homologs are highly conserved across plant species in negatively regulating cell death and immunity. Given that cell death occurs in *bir1-1* mutants even under sterile conditions, BIR1 may not function in perception of PAPMs (pathogen-associated molecular patterns).

### 3.2. The Constitutively Activated Cell Death and Defense Responses in GmBIR1-Silenced Plants Is A Result of Co-Silencing of Both GmBIR1a and GmBIR1b

Soybean is a paleopolyploid and 75% of genes in its genome contain at least two copies [39]. Consistent with this, there are two *GmBIR1* genes, *GmBIR1a* and *GmBIR1b,* in the soybean genome. The open reading frame sequences of *GmBIR1a* and *GmBIR1b* share 95% identity at the nucleotide level. The DNA fragment used for *GmBIR1* silencing contains four stretches of sequences with 100% identity between *GmBIR1a* and *GmBIR1b* that could potentially generate siRNAs of >20 nt in length (Supplementary Figure S1). Therefore, both *GmBIR1a* and *GmBIR1b* were silenced using the same construct (Figure 1G). The autoimmune phenotype of the *GmBIR1*-silenced plants (Figure 1B) actually is a result of co-silencing of both *GmBIR1a* and *GmBIR1b*. We have previously shown that multiple copies of *GmMPK4, GmMPK6*, *GmMEKK1*, *GmFLS2*, and *GmATG2* genes could be successfully co-silenced using a single BPMV-VIGS construct [20,22,23,24,25]. Given that soybean is polyploidy and is recalcitrant to transformation, VIGS is currently one of the most efficient approaches to interrogate gene functions in soybean [26,27].

### 3.3. Does the Autoimmunity Observed in GmBIR1-Silenced Plants Result from Activated PTI or Activated ETI?

Activation of either PTI or ETI results in autoimmune responses, although the autoimmune responses resulting from ETI are much stronger and last longer than those resulting from PTI [1]. Structure analysis indicates that Arabidopsis BIR1 and BAK1 directly interact with each other through their ecto-LRR domains [36]. Under no-elicited conditions, BIR1^LRR^–BAK1^LRR^ interaction sequesters BAK1 and prevents formation of a signal competent complex with FLS2. In the presence of flg22, flg22-bound FLS2^LRR^ outcompetes BIR1^LRR^ for binding to BAK1^LRR^ from the BIR1^LRR^–BAK1^LRR^ complex and forms a signaling competent flg22–FLS2^LRR^–BAK1^LRR^ complex [36] Based on this structural study, BIR1 functions to keep BAK1-mediated cell death signaling under tight control, thus preventing undesired autoimmunity, and therefore, it is possible that the autoimmunity in *bir1-1* or *GmBIR1*-silenced plants is a result of activation of BAK1-mediated PTI. Our *Psg* infection assay supported this possibility (Figure 4). However, inconsistent with this possibility, the flg22-induced activation of *Gm*MPK3 and *Gm*MPK6, which is a hallmark event of activated PTI (Meng et al., 2013), was significantly reduced in *GmBIR1*-silenced plants relative to vector control plants (Figure 4), and the activation of MPK3 and MPK6 in the Arabidopsis *bir1-1* mutant is unchanged compared to WT Col-0 [14]. Therefore, it is still unclear whether the autoimmune responses observed in *bir1-1* or *GmBIR1*-silenced plants are solely due to activated PTI, reflecting the complexity of the involvement of BIR1 in plant immunity.

Several lines of evidence indicate that autoimmune responses in *bir1-1* or the *GmBIR1*-silenced plants could result from misactivation of R proteins. Firstly, loss function of *BIR1* in Arabidopsis leads to the impairment in the activation of MPK4 by flagellin [14]. The autoimmune phenotype of *bir1-1* could be the consequence of the impaired MPK4 activation given that Arabidopsis MEKK1-MKK1/2-MPK4 signaling module negatively regulates the activation of the R proteins SUMM2 and SMN1/RPS6 [40,41,42]. Misactivation of SUMM2 and SMN1/RPS6 result from mutations in MEKK1-MKK1/2-MPK4 pathway leads to autoimmune responses [40,42]. However, *Gm*MPK4 activity cannot be induced either by flg22 or SA, regardless of concentration and duration of treatments [23]. Therefore, it is unclear whether *Gm*MPK4 plays a similar role as its counterpart in Arabidopsis. As the *Gm*MPK4 activation is also not observed in the vector control plants (Figure 4 and [23]), it is unlikely that the *GmBIR1*-silenced phenotypes are related to *Gm*MPK4 function. Secondly, in *Arabidopsis thaliana*, the copain-like protein BONZAI1 (BON1) interacts physically with and phosphorylates BIR1 and *bir1-1* mutant is partially suppressed by loss of function mutation in the resistance gene *SNC1* [18]. Thirdly, the autoimmune phenotype of *bir1-1* can be partially suppressed by elevated temperatures [18], which is a characteristic feature of R protein-mediated responses [43,44]. Lastly, the autoimmune phenotype of *bir1-1* is rescued by the loss of function of EDS1 and PAD4 [14], two key players in TIR type of R protein activation [45]. These results strongly suggest that the autoimmune phenotype of the *bir1-1* mutant is a result of misactivated R protein(s). Therefore, it is possible that BIR1 might participate in both PTI and ETI through interacting with different proteins, and the autoimmune responses observed in *bir1-1* and *GmBIR1*-silenced plants could be combined effects of activated PTI and ETI. The facts that RLKs/RLPs are required for NLR-mediated immunity [46] and RLKs/RLPs and NLRs mutually potentiate each other [47] support the idea that BIR1 may participate in both PTI and ETI.

### 3.4. Is the Enhanced Resistance of GmBIR1-Silenced plants to SMV SA-Dependent?

The enhanced resistance of *GmBIR1*-silenced plants against *Psg* could be attributed to the increased accumulation of both SA and H_2_O_2_ (Figure 2) and is thus SA-dependent. The enhanced SMV resistance in *GmBIR1*-silenced plants may not be solely due to increased accumulation of both SA and H_2_O_2_. In Arabidopsis, Guzman-Benito et al. [38] reported that the enhanced resistance of *bir1-1* to *Tobacco rattle virus* (TRV) is independent of cell death or SA defense priming. The expression of Arabidopsis *BIR1* is regulated both at TGS and PTGS level by RNA silencing pathway [38]. A threshold level of *BIR1* expression must be properly maintained and an expression beyond the threshold level (either too high or too low) triggers autoimmunity. Whether the enhanced resistance of *GmBIR1*-silenced plants to SMV is uncoupled from the cell death needs to be further investigated. 

## 4. Materials and Methods

### 4.1. Plant Materials

Seeds of soybean (Glycine max ‘Williams 82’) used in this study were harvested from greenhouse-grown plants previously indexed for the absence of BPMV and SMV [48,49]. Soybean plants were grown in a growth chamber at 22 degrees with a photoperiod of 16 h.

### 4.2. Flg22 Peptides

The flg22 peptide was synthesized by HuaBio (Hangzhou, China).

### 4.3. BPMV-Mediated VIGS

BPMV strains, BPMV VIGS constructs, and inoculation of soybean seedlings with DNA-based BPMV constructs via biolistic particle bombardments using a Biolistic PDS-1000/He system (Bio-Rad Laboratories, Hercules, CA, USA) have been described previously [48]. The orthologs of ArabidopsisBIR1 in the soybean genome were identified by BLASTN in phytozome databases. The primers used for construction of the BPMV-*GmBIR1* (Glyma.18g246400.1) are:

*GmBIR1*-F: 5′-aagGGATCCCTCACTGGTCAAATTCCTGCTA-3′
*GmBIR1*-R: 5′-ttgGGTACC**C**AGGGTCCTCTTCCTTCTTCCTA-3′

The underlined sequences are *Bam*H I and *Kpn* I restriction sites, respectively, which were used for directional cloning of the PCR fragment into the BPMV VIGS (IA-D35). The letter C in bold indicates an extra nucleotide in reverse primers needed to maintain the correct open reading frame with BPMV genome.

### 4.4. RNA Isolation and RT-PCR

RNA isolation and RT-PCR were performed as described elsewhere [50]. The RNA samples extracted were treated with DNaseI according to the manufacturer’s instructions (Invitrogen, Carlsbad, CA, USA). Primers used for RT-PCR in this study are:

*GmELF1b*-F: 5′- ACCGAAGAGGGCATCAAATCCC -3′
*GmELF1b*-R: 5′- CTCAACTGTCAAGCGTTCCTC -3′
*GmBIR1*-full-F: 5′- ATAGGAAGAAGGAAGAGGACC -3′
*GmBIR1*-full-R: 5′- TTTAGGAGTAGCCACCAAAGT -3′
*GmBIR1a-F*: 5′- GACTTGCTCAATATCCCAGGATAAGT-3′
*GmBIR1a-R*: 5′- TGTTGAGATTCCTGTAATCTCTTAACCAT -3′
*GmBIR1b-F*: 5′- TACTTCCTCAATATCCCAGGTGG -3′
*GmBIR1b-R*: 5′- TATTGAGATTCCTGCAATCTCTTGAC -3′.

The *GmBIR1a*-full primers were used for amplifying the full-length cDNA and the *GmBIR1a* and *GmBIR1b* primers are used for determining the specific transcript levels of the *GmBIR1a* and *GmBIR1b,* respectively.

### 4.5. Trypan Blue Staining

Cell death was detected specifically by trypan blue staining solution as described by [18]. At 18 dpi with BPMV vector only (BPMV-0) or BPMV-*GmBIR1* constructs, second fully expanded soybean trifoliolate leaves counting from the top were detached. The detached leaves were placed in trypan blue staining solution (10 mL glycerol, 10 mL lactic acid, 10 mL water, 10 mg trypan blue powder and 10 g phenol) and boiled for 1–1.5 min. The stained leaves were cleared using tap water and then dipped in the chloral hydrate solution in the shaker overnight to remove the non-specific stains. Images were captured using a stereo microscope (Olympus SZH10, Center Valley, PA, USA).

### 4.6. SA Quantification

SA was quantified using an Agilent 1260 HPLC system (Agilent Technologies) with a diode array detector and a fluorescence detector and a column as described previously [51]. The column was a 4.6-by-75-mm Zorbax SB-C18 with a mobile phase comprising sodium acetate (0.2 m, pH 5.5) and methanol with a series of concentration gradients. The initial methanol gradient was maintained at 3% (*v*/*v*) for 12 min, linearly increased to 7% (*v*/*v*) at 12.5 min and maintained until 38 min at a flow rate of 0.8 mL min−1 throughout the process. After 1 min, the initial reaction conditions were restored and the system needed to be equilibrated for 7 min before the next injection. Total SA from the sample treated with β-Glucosidase and free SA were detected with a 296 nm excitation wavelength and 410 nm emission wavelength. The concentration was calculated by determining the HPLC peak area according to a standard curve.

### 4.7. H_2_O_2_ Detection by DAB Staining

H_2_O_2_ was detected by an endogenous peroxidase-dependent in situ histochemical staining procedure using DAB (Sigma-Aldrich, St. Louis, MO, USA) [32]. Detached leaves were placed in a solution containing 1 mg mL−1 DAB (pH 5.5) for 2 h. The leaves were cleared by boiling in 96% (*v*/*v*) ethanol for 10 min. H_2_O_2_ production was visualized as a reddish-brown precipitate on the cleared leaves [32].

### 4.8. Inoculation of Pseudomonas Syringae pv. Glycinea (Psg) onto the Leaves of Soybean Plants

*Psg* growth assay was performed as described by [24]. *Psg* was cultivated at 28 degrees until OD = 1.3. The bacterial culture was centrifuged at 3000 rpm for 10 min and the pellet was re-suspended in double-distilled water with an OD = 1. The vector control or *GmBIR1*-silenced plants were thoroughly and evenly sprayed with the re-suspended bacterial solution on both upsides and downsides of the leaves. The sprayed plants were immediately covered with transparent plastic bags for at least 24 h.

### 4.9. SMV-N-GUS Inoculation, GUS Staining, and GUS Foci Measurements

At 18 dpi with BPMV vector only (BPMV-0) or BPMV-*GmBIR1* constructs, second fully expanded soybean trifoliolate leaves counting from the top were detached and biolistically inoculated with SMV-N-GUS [33,48]. Following SMV-N-GUS inoculation, the detached leaves were put into petri dishes with moist filter papers and kept on a lighted growth shelf for 5 days before GUS staining. GUS staining was performed as described by [52]. Photographs of the leaves with GUS foci were taken using a stereo microscope (Olympus SZH10, Center Valley, PA, USA). The numbers of GUS foci were counted, and the diameters of GUS foci were measured using Soft Image System analysis (IA Package; Olympus).

### 4.10. Western Blot Analysis for Detecting Phosphorylated MPKs

Protein was extracted from soybean leaf tissues using extraction buffer (50 mM Tris-MES pH 8.0, 0.5 M sucrose, 1 mM MgCl_2_, 10 mM EDTA, 5 mM DTT) with protease inhibitor cocktail S8830 (Sigma-Aldrich, St. Louise, MO, USA) added as described by [23]. The extract was centrifuged at 12,000 rpm at 4 °C for 30 min and the supernatant was collected. For immunoblotting, the extracted proteins were separated by SDS-PAGE (10% acrylamide gel) and transferred to PVDF membrane (Millipore, Billerica, MA, USA) by semi-dry electro-transfer (Bio-Rad, Hercules, CA, USA). The membrane was blocked in 1× TBST buffer containing 5% milk powder for 2 h. After washing, the membrane was further incubated with anti–Phospho-p44/p42 MAPK (anti-pTEpY) diluted at 1:3000 (Cell Signaling Technology, Danvers, MA, USA), followed by incubation with secondary antibody diluted at 1:7500. The bands were visualized by incubating with chemiluminescent HRP substrate (Millipore, Billerica, MA, USA).

### 4.11. Subcellular Localization of GmBIR1

The *GmBIR1* (Glyma.18g246400.1) open reading frame was amplified by RT-PCR from total RNA extracted from cv Williams 82 soybean plants using the following primers:

*Gm*BIR1-PGDG-F-HinDIII: 5′-**AAGCTT**ATGAAGATGTTTATGGGTGGC-3′
*Gm*BIR1-PGDG-R-BamHI: 5′-**GGATCC**TCAATCATGTCCCTCTCGAG-3′

The letters in bold represent the cleavage sites for different restriction enzymes.

The RT-PCR products were firstly double digested with HindIII + BamHI and subsequently cloned into the PGDG vector [53] to generate the GFP-*Gm*BIR1 fusion construct. These fusion constructs and the *AtCBL1*-mCherry construct (membrane marker) were coinfiltrated into *N. benthamiana* leaves as described by [50]. Images were captured with a confocal laser-scanning microscope (Leica TCS SP5 AOBS, Wetzlar, Germany).

## Data Availability

No applicable.

## Supplementary Materials

The following supporting information can be downloaded at: https://www.mdpi.com/article/10.3390/ijms23137450/s1.
