# Supplementary material for: Silencing GmBIR1 in Soybean Results in Activated Defense Responses
Source: Int J Mol Sci. 2022 Jul 5;23(13):7450. doi: 10.3390/ijms23137450 (PMC9267208; doi:10.3390/ijms23137450)
Supplement: Supplementary file 1 [file ijms-23-07450-s001.zip › ijms-1783824-supplementary.pdf]

```
GmBIR1a CTCACTGGTCAAATTCCTGCTAACTTGAGTCAGCTCCCGCGGCTTAAGTTGTTTAGTGTT
        ||||| ||||| |||||||| |||||||||||||||||||||||||||||||||||||||
GmBIR1b CTCACCGGTCACATTCCTGCAAACTTGAGTCAGCTCCCGCGGCTTAAGTTGTTTAGTGTT

GmBIR1a GCCAATAATCTTTTGACAGGGCAAGTTCCAATCTTTGCAAATGGTGTAGCTAGTGCCAAT
        |||||||||||||||||||||| |||||||  ||||  |  |||||||||| ||||  ||
GmBIR1b GCCAATAATCTTTTGACAGGGCCAGTTCCACCCTTTAAACCTGGTGTAGCTGGTGCAGAT

GmBIR1a AGTTATGCAAATAATTCAGGTCTGTGTGGTAAACCCTTATTGGATGCTTGCCAGGCCAAG
        | |||||||||||||||||| ||||||||||| |||   |||| | ||||||||| |  |
GmBIR1b AATTATGCAAATAATTCAGGCCTGTGTGGTAATCCC...TTGGGTACTTGCCAGGTCGGG

GmBIR1a GCTTCGAAGAGTAACACAGCTGTTATAGCTGGAGCAGCTGTTGGTGGTGACTGTTGCA
         |||| ||||||||||| ||||||||||||||||||||||||||||||||||||||||
GmBIR1b TCTTCAAAGAGTAACACTGCTGTTATAGCTGGAGCAGCTGTTGGTGGTGACTGTTGCA

GmBIR1a GCATTAGGTTTGGGCATTGGAATGTTCTTCTACGTGCGCCGTATTTCTTATAGGAAGAAG
        ||||||||||||||||||||||||||||||||||||||||||||||||||||||||||||
GmBIR1b GCATTAGGTTTGGGCATTGGAATGTTCTTCTACGTGCGCCGTATTTCTTATAGGAAGAAG

GmBIR1a GAAGAGGACCCT
        ||||||||||||
GmBIR1b GAAGAGGACCCT
```

Supplemental Figure S1. Comparison of *GmBIR1a* sequence used for silencing vector construction with the corresponding sequence of *GmBIR1b*.
